# Pulmonary Hypertension and Acute Heart Failure Following Lumbar Disc Surgery

**DOI:** 10.1016/j.jscai.2024.102453

**Published:** 2024-12-24

**Authors:** Bárbara Lacerda Teixeira, André Grazina, Luís Almeida Morais, Ana Galrinho, Rui Cruz Ferreira

**Affiliations:** Hospital Santa Marta – Centro Hospitalar Universitário Lisboa Central, Lisbon, Portugal

**Keywords:** arteriovenous fistula, heart failure, high cardiac output, pulmonary hypertension

## Abstract

A 70-year-old woman was admitted with heart failure and signs of pulmonary hypertension on echocardiogram. Cardiac catheterization demonstrated postcapillary pulmonary hypertension, elevated cardiac output, and O_2_ saturation enrichment. Computed tomography revealed a large arteriovenous fistula between the common iliac artery and vein, which was due to a lumbar disc surgery performed several years prior.

## Case description

A 70-year-old woman with a history of hypertension, rheumatoid arthritis, cholecystectomy, and L4-L5 laminectomy was admitted because of progressive heart failure (HF) with severe dyspnea, pleural effusion, and pitting edema. A systolic murmur was detected on cardiac auscultation. Electrocardiogram was unremarkable, and there was elevation of NT-proBNP at 9181 pg/mL. Transthoracic echocardiogram was suggestive of severe pulmonary hypertension (PH) with right ventricle dilatation and D-shaped septum, pulmonary artery dilatation, moderate tricuspid valve regurgitation, systolic pulmonary artery pressure of 69 mm Hg, and mild pericardial effusion. Transesophageal echocardiogram excluded intracardiac shunts.

Computed tomography (CT) angiography excluded pulmonary embolism or lung disease, detecting marked dilatation of the inferior vena cava and iliac veins, but did not report any vascular communication. Right heart catheterization confirmed PH with a mean pulmonary artery pressure of 32 mm Hg and elevation of pulmonary capillary wedge pressure at 20 mm Hg. Pulmonary vascular resistance and diastolic pulmonary gradient were normal. The cardiac output and cardiac index, determined using the Fick method, were severely elevated at 10.96 L/min and 7.88 L/min/m^2^, respectively. Blood gas analysis revealed O_2_ saturation enrichment in the venous system until the left common iliac vein. The right heart catheterization results are shown in Supplementary Table S1.

**Figure 1:**
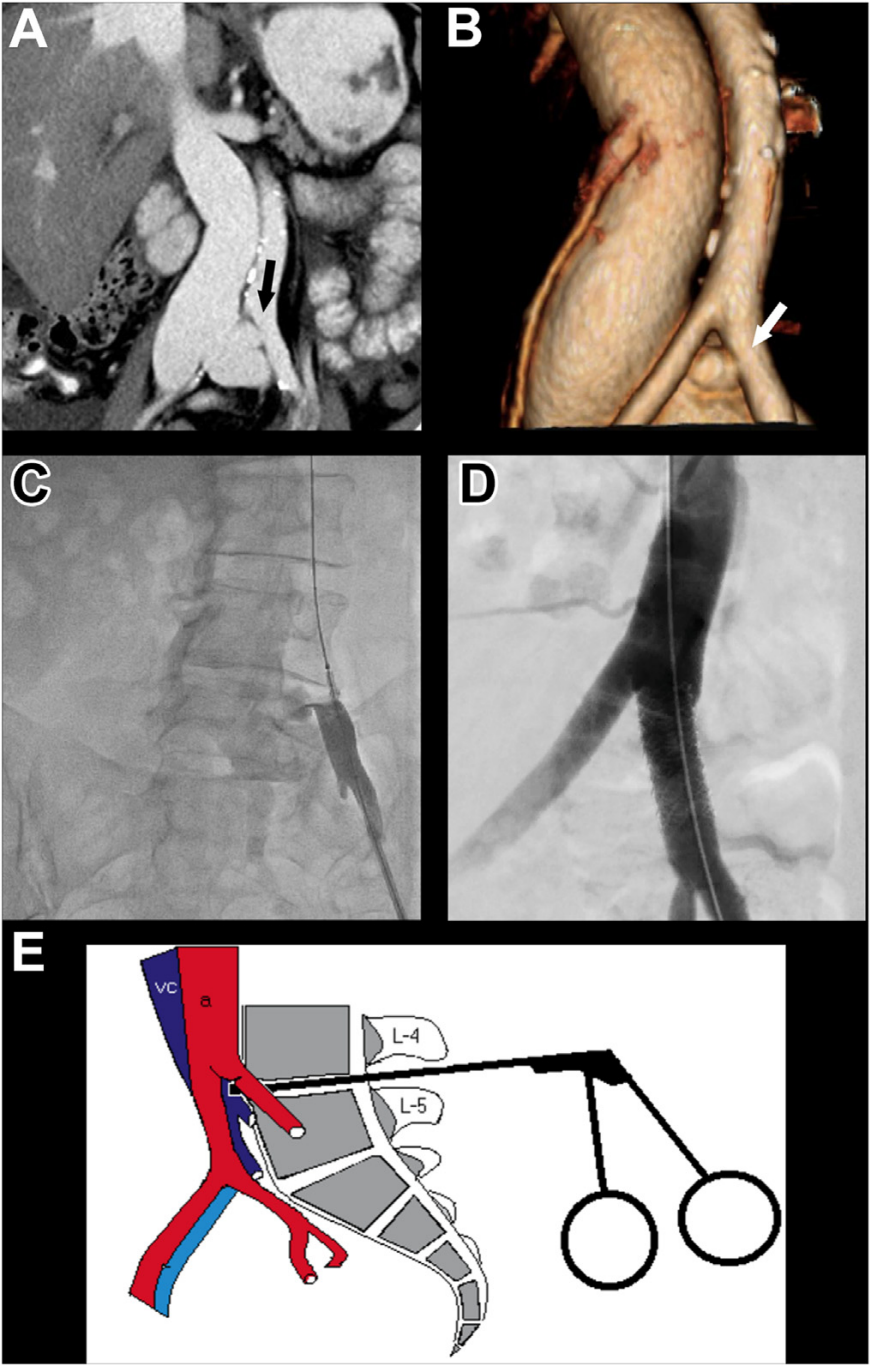
**Iliac arteriovenous fistula—diagnosis and management.** (A) Computed tomography angiography (CTA) showing enlargement of the inferior vena cava compared with the aorta and an arteriovenous (AV) fistula between the left common iliac artery and the ipsilateral vein (arrow). (B) 3D reconstruction of the CTA scan shown in (A) and the AV fistula (arrow). (C) Aortography identifying the fistula and a large contrast shunt between the left common iliac artery and vein. (D) Aortography after closure of the AV fistula with a 9 × 32 mm Advanta covered stent. (E) Anatomy of the iliac vessels in relation to the L4-L5 intervertebral disc and laminectomy. From Papadoulas et al,^1^ with permission of the author.

In light of these hemodynamic findings and saturation run, an extracardiac shunt localized near the left common iliac vein was evident. After revision of CT scans, a 10 × 7 mm arteriovenous (AV) fistula between the left common iliac artery and the ipsilateral vein was identified (Figure 1A, B).^1^ The patient underwent aortography, which identified a large shunt between the left common iliac artery and vein (Figure 1C). A 7F sheath was placed in the left common femoral artery, and a wire was retrogradely placed in the aorta. The team proceeded to close the AV fistula with a 9 × 32 mm Advanta covered stent (Getinge) that was placed at the left common iliac artery (Figure 1D) and postdilated with a 10-mm balloon, with great angiographic result and no signs of fistula (Supplementary Videos 1-5). The source of the fistula was the laminectomy, performed 4 years prior to the onset of the symptoms of HF.

After the closure of the AV fistula, transthoracic echocardiogram showed reduction of the estimated systolic pulmonary artery pressure to 26 mm Hg and less dilatation of the right chambers. Diuretics were reduced, HF symptoms improved greatly, and NT-proBNP at discharge was 743 pg/mL. At the 1-year follow-up, the patient is asymptomatic and without signs of HF.

## Discussion

Although uncommon, high cardiac output HF is often associated with a potentially correctable etiology, namely chronic anemia, systemic AV fistula, sepsis, hypercapnia, and hyperthyroidism.^2^ The echocardiogram signs of right ventricle pressure overload made us prioritize the exclusion of PH with invasive methods. This case was rather challenging, because AV shunts have long been known to potentially cause high output HF,^3^ but our patient did not appear to have any obvious cause. Settling the hemodynamic changes invasively helped us narrow the differential diagnosis, despite the initial undiagnostic CT. In our case, an intracardiac shunt was excluded by transesophageal echocardiogram and cardiac catheterization. When facing PH with normal pulmonary vascular resistance and diastolic pulmonary gradient, elevated cardiac output, and O_2_ saturation enrichment, an extracardiac shunt should be suspected.^3^

AV fistulas can be congenital or acquired. The latter may be iatrogenic, such as in our patient, or due to trauma.^2^ Our patient had 2 prior surgeries, and we believe the lumbar disc surgery in the L4-L5 intervertebral disc was the cause of the fistula, evidenced by anatomic relations (Figure 1E).^1^ Although rare, the literature reports 1 to 5 vascular complications per 10,000 lumbar disc operations. Lacerations are more commonly observed and are rapidly identified in the postoperative period. On the contrary, AV fistulas are rarer and can be undiagnosed for a long time, and they may present as congestive HF, as in our case. The ideal treatment is excision or correction of the fistula, whether by surgery or endovascular treatment.^1^

## Supplementary material

Supplementary Table S1 Right heart catheterization results, including hemodynamic variables and saturation run values. To calculate diastolic pulmonary gradient, left ventricle end-diastolic pressure was used instead of pulmonary artery wedge pressure.

Supplementary Video 1 Aortography identifying a large contrast shunt between the arterial and venous systems.

Supplementary Video 2 Aortography specifically identifying the fistula and contrast shunt between the left common iliac artery and vein.

Supplementary Video 3 Closure of the arteriovenous fistula with a 9 × 32 mm Advanta covered stent.

Supplementary Video 4 Results after the closure with stent implantation, without any signs of fistula.

Supplementary Video 5 Final aortography after closure, without any signs of fistula.
