# Supplementary material for: Pulmonary Hypertension and Acute Heart Failure Following Lumbar Disc Surgery
Source: J Soc Cardiovasc Angiogr Interv. 2024 Dec 24;4(1):102453. doi: 10.1016/j.jscai.2024.102453 (PMC11887551; doi:10.1016/j.jscai.2024.102453)
Supplement: Supplementary Table S1 — Right heart catheterization results, including hemodynamic variables and saturation run values. To calculate diastolic pulmonary gradient, left ventricle end-diastolic pressure was used instead of pulmonary artery wedge pressure. [file mmc1.docx]

| **Hemodynamic variable** | |
| --- | --- |
| Right ventricular pressure (systolic / diastolic) in mmHg | 60 / 12 mmHg |
| Pulmonary artery pressure (systolic / diastolic / mean) in mmHg | 60 / 20 / 32 mmHg |
| Pulmonary vascular resistance in WU | 1.1 WU |
| Cardiac Output in L/min (Fick) | 10.96 L/min |
| Cardiac Index in L/min/m2 (Fick) | 7.88 L/min/m^2^ |
| Right atrium pressure (mean) in mmHg | 12 mmHg |
| Pulmonary artery wedge pressure (mean) in mmHg | 20 mmHg |
| Left ventricle end-diastolic pressure (mean) in mmHg | 18 mmHg |
| Diastolic pulmonary gradient in mmHg | 2 mmHg |
| Pulmonary artery pulsatility index | 3.3 |
| Arterial (aorta) saturation in % | 97% |
| **Venous saturation in %** | |
| Right subclavian vein | 42% |
| Superior Vena Cava | 55% |
| Right Atrium | 82% |
| Right Ventricle | 81% |
| Pulmonary Artery | 83% |
| Inferior Vena Cava | 93% |
| Left Common Iliac Vein | 93% |
| Right Common Iliac Vein | 71% |

**Supplementary Table 1.** Right heart catheterization results, including hemodynamic variables and saturation run values. To calculate diastolic pulmonary gradient, it was used left ventricle end-diastolic pressure instead of pulmonary artery wedge pressure.
